# Down to the Wire: A Case of Gastrointestinal Bleeding After Splenic Artery Coiling

**DOI:** 10.14309/crj.0000000000000835

**Published:** 2022-07-21

**Authors:** John P. Haydek, Augustin R. Attwell

**Affiliations:** 1 Division of Gastroenterology and Hepatology, Department of Medicine, University of Colorado, Aurora, CO; 2 Denver Health Medical Center, Denver, CO

## CASE REPORT

**Figure 1.**
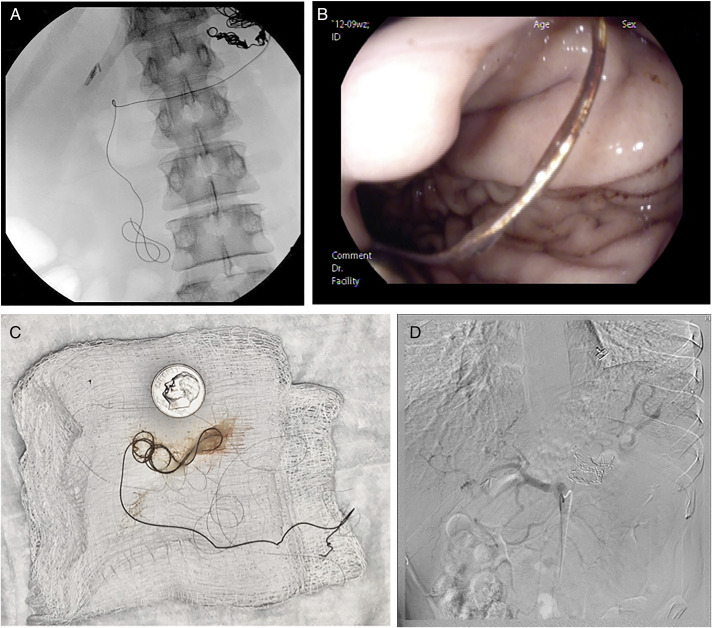
(A) Fluoroscopic image of extravasating wire. (B) Endoscopic image of wire extending into lumen. (C) Photograph of wire, with coin for scale. (D) Fluoroscopic angiogram showing a lack of ongoing bleeding following wire removal.

A 36-year-old man with a history of alcoholic pancreatitis complicated by splenic artery pseudoaneurysm treated with angiographic coiling 18 months earlier presented with worsening abdominal pain and new hematemesis. X-ray of the abdomen showed unraveling and migration of one of the splenic artery coils into the gastroduodenal lumen (Figure 1). Esophagogastroduodenoscopy showed the coil protruding into the gastric lumen (Figure 1) with slow bleeding at the entry site and extension of the coil into the distal duodenum without additional injuries. A contrast computed tomography scan showed no intra-abdominal bleeding. With interventional radiology colleagues present in the endoscopy suite and using fluoroscopy, the exposed coil was grasped and removed slowly using standard biopsy forceps (Boston Scientific, Marlborough, MA) while verifying that the remaining intravascular coils did not move or migrate (Figure 1). After coil removal, slow but persistent oozing was noted from the gastric entry site, so an over-the-scope clip (Ovesco Endoscopy, Tuebingen, Germany) was deployed over this area resulting in immediate cessation of bleeding. The patient underwent repeat angiography with coiling of a gastric artery branch to prevent intra-abdominal bleeding (Figure 1D). He did well subsequently without further gastrointestinal (GI) bleeding and was discharged from the hospital within a week. He has not had further intra-abdominal or luminal bleeding since the esophagogastroduodenoscopy 6 months ago.

Endovascular coil migration into the GI tract is a rare complication after vascular embolization, and these events may cause GI or intra-abdominal bleeding.^1^ Although there are little data to guide decision making, management has generally depended on the patient's presentation and hemodynamic stability. Observation is generally preferred for asymptomatic patients, but endoscopy and exploratory laparotomy have been performed when bleeding or pain results.^2–5^

Our case is unique in several aspects. First, other reported cases of intraluminal coil migration do not routinely perform extraction of the exposed coil, which we accomplished using biopsy forceps. Second, we used an over-the-scope clip for prevention of bleeding from the site of the retained coil, which is a new technique. Finally, the procedure was technically and clinically successful as of the 6-month follow-up.

## DISCLOSURES

Author contributions: JP Haydek reviewed the literature, wrote the manuscript, revised the manuscript, and approved the final manuscript. AR Attwell provided images, reviewed the manuscript, provided final approval for the article, and is the article guarantor.

Financial disclosure: None to report.

Informed consent was obtained for this case report.

## References

[1] Skipworth JR Morkane C Raptis DA . Coil migration—A rare complication of endovascular exclusion of visceral artery pseudoaneurysms and aneurysms. Ann R Coll Surg Engl. 2011;93(4):e19–23. 21944789 10.1308/003588411X13008844298652 PMC5827005

[2] Palagiri AN Hamo F Samo S Chawla S. Coil erosion into the duodenum following arterial embolization. ACG Case Rep J. 2019;6(8):e00195. 31737725 10.14309/crj.0000000000000195 PMC6791628

[3] Ly M Brown K Coker D Rice M. Endovascular coil erosion into the duodenum following gastroduodenal artery angioembolization. ANZ J Surg. 2020;90(9):1816–8. 32053249 10.1111/ans.15715

[4] Han YM Lee JY Choi IJ . Endoscopic removal of a migrated coil after embolization of a splenic pseudoaneurysm: A case report. Clin Endosc. 2014;47(2):183–7. 24765602 10.5946/ce.2014.47.2.183 PMC3994262

[5] Pratap A Pokala B Vargas LM Oleynikov D Kothari V. Laparoscopic endoscopic combined surgery for removal of migrated coil after embolization of ruptured splenic artery aneurysm. J Surg Case Rep. 2018;2018(2):rjx242. 29479413 10.1093/jscr/rjx242 PMC5810436

